# The complete mitochondrial genome of nematophagous fungus *Esteya vermicola*

**DOI:** 10.1080/23802359.2017.1307700

**Published:** 2017-04-01

**Authors:** Ruizhen Wang, Leiming Dong, Yuequ Chen, Liangjian Qu, Enjie Li, Qinghua Wang, Yongan Zhang

**Affiliations:** a The Key Laboratory of Forest Protection, State Forestry Administration of China, Research Institute of Forest Ecology, Environment and Protection, Chinese Academy of Forestry, Beijing, China;; b State Key Laboratory of Tree Genetics and Breeding, Research Institute of Forestry, Chinese Academy of Forestry, Beijing, China;; c Forestry Resources Protection Institute, Jilin Provincial Academy of Forestry Sciences, Changchun, China

**Keywords:** Mitochondrial genome, *Esteya vermicola*, phylogenetic analysis

## Abstract

The complete mitochondrial genome of the Nematophagous fungus *Esteya vermicola* CBS 115803 was determined using the PacBio RS II sequencing technology. The circular molecule is 47,282bp in length with a GC content of 24.85%. Annotated genes including 14 conserved protein-coding genes, the large and the small rRNA subunit (rnl and rns) and 27 tRNAs. The phylogenetic analysis showed that *E. vermicola* had close genetic relationship with the genus *Sporothrix*.

*Esteya vermicola* is known as the recorded endoparasitic nematophagous fungus that attacks pine wood nematode (PWN, *Bursaphelenchus xylophilus*) (Liou et al. 1999). Adhesive lunate conidia produced by *E. vermicola* can attach to and penetrate the cuticle of PWN and consume contents of the infected nematode’s body, and then kill nematode and produce new lunate conidia for the next infection cycle (Wang et al. 2011, 2010, 2008). Up to now, there are six strains of *E. vermicola* around the world (Chu et al. 2015). However, the complete mitochondrial genome of *E. vermicola* has not been reported. Sequencing the complete mitochondrial genome of *E. vermicola* will enhance our understanding of the evolutionary relationships between *E. vermicola* and its related closed species.

The strain of *E. vermicola* CBS 115803 was originally isolated from *Scolytus intricatus* and its galleries in oak trees in Czech Republic (Chu et al. 2015) and was stored in the CBS-KNAW culture collection in the Netherlands. Here, we present the complete mitochondrial genome of *E. vermicola* CBS 115803 (GenBank accession number: KY644696). Total genomic DNA was extracted using Genomic-tip Kit 500G (Cat No./ID: 10262) following the manufacturer’s instructions, then was build up 20kb genomic library and sequenced using PacBio RS II platform. Sequencing depth was 104.66 × coverage. Mitochondrial reads of *E. vermicola* were filtered depending on the database of fungal mitogenome, and assembled using HGAP2.3.0 (SMRT Analysis). The mitochondrial genome of *E. vermicola* was assembled as a 47,282bp single circular molecule, which encoded 16 genes including three cytochrome oxidases (*cox*), seven subunits of NAD dehydrogenase (*nad*), the large and the small rRNA subunit (*rnl* and *rns*), three ATP synthases (*atp*), and one cytochrome b (*cob*).

A total of 27 tRNA genes were predicted in the mitochondrial genome of *E. vermicola* corresponding to 21 amino acids using tRNAscan software, including 19 common amino acids tRNA (three tRNA-Met), extraordinary 21st selenocysteine tRNA which takes part in proteins involved in antioxidant activity (Byun & Kang 2011) and one suppressor tRNA. The base composition of mitochondrial genome of *E. vermicola* is 38.08% A, 37.06% T, 14.05% G, and 10.81% C, with a low GC content of 24.85%. The gene order of all conserved protein-coding genes and tRNA was *Cys*, *Arg*, *cox1*, *nad1*, *nad4*, *atp8*, *atp6*, *rns*, *Tyr*, *Asn*, *cox3*, *Lys*, *Asp*, *Ser*, *SeC*, *nad6*, *Sup*, *Val*, *Ile*, *Ser*, *Pro*, *rnl*, *Lys*, *Thr*, *Glu*, *Met*, *Met*, *Leu*, *Gly*, *Ala*, *Phe*, *Leu*, *Gln*, *His*, *Met*, *nad2*, *nad3*, *atp9*, *cox2*, *Arg*, *nad4L*, *nad5*, *cob*.

To validate the phylogenetic position of *E. vermicola*, phylogenetic analyses using maximum-likelihood (ML) method were conducted in MEGA7 using GTR + G + I model with 1000 bootstrap replicates (Figure 1). 15 species were used to construct phylogenetic tree, and they are *Beauveria bassiana* D1-5 mit, *Cordyceps bassiana* isolate Bb147, *B. pseudobassiana* NC 022708.1, *C. militaris* strain EFCC-C2, *Metarhizium anisopliae* strain ME1, *Hirsutella minnesotensis* strain 3608, *H. rhossiliensis* isolate USA-87-5, *Fusarium graminearum* strain CBS 110263, *Aspergillus nidulans* FGSC A4, *Neurospora crassa* OR74A, *Esteya vermicola* CBS115803, *Sporothrix brasiliensis* 5110 Cont23, *S. schenckii* KMU2052, *S. schenckii* ATCC 10268, and *S. schenckii* NC 015923.1. Phylogenetic analysis based on whole mitochondria genome sequences clustered *E. vermicola* with the genus *Sporothrix*, so it confirms *E. vermicola* has close genetic relationship with the genus *Sporothrix* according to phylogenetic analysis (Figure 1).

**Figure 1.**
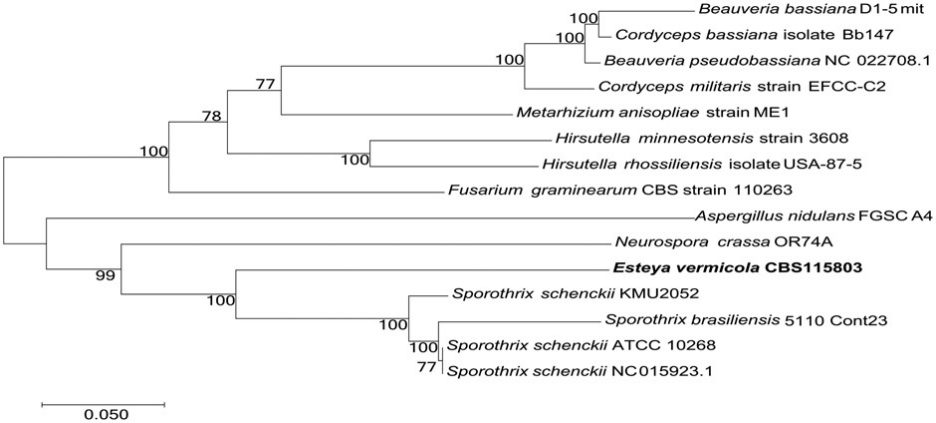
Phylogenetic relationships (maximum likelihood) of *Esteya vermicola* based on the nucleotide sequence of 14 conserved protein-coding genes (atp6, atp8, atp9, cob, cox1, cox2, cox3, nad1, nad2, nad3, nad4, nad4L, nad5, nad6) in the mitochondrial genomes. The numbers beside the nodes are percentages of 1000 bootstrap values.
